# Effect of rapid weight loss incorporating hot salt water immersion on changes in body mass, blood markers, and indices of performance in male mixed martial arts athletes

**DOI:** 10.1007/s00421-022-05000-7

**Published:** 2022-07-14

**Authors:** John Connor, Mark Germaine, Conor Gibson, Philip Clarke, Brendan Egan

**Affiliations:** 1 School of Health and Human Performance, Dublin City University, Glasnevin, Dublin 9, Ireland; 2 National Institute for Cellular Biotechnology, Dublin City University, Dublin, Ireland; 3 Florida Institute for Human and Machine Cognition, Pensacola, FL USA

**Keywords:** Body water, Fluid balance, Heat, Hydration, Magnesium, Sweat

## Abstract

**Purpose:**

To investigate the effects of rapid weight loss (RWL), incorporating comparison of hot water immersion (HWI) in fresh or salt water, on changes in body mass, blood markers, and indices of performance in mixed martial arts athletes.

**Methods:**

In a crossover design comparing fresh water (FWB) to salt water (SWB; 5.0%wt/vol Epsom salt) bathing, 13 males performed 20 min of HWI (~ 40.3 °C) followed by 40 min wrapped in a heated blanket, twice in sequence (2 h total). Before bathing, ~ 26 to ~ 28 h of fluid and dietary restriction was undertaken, and ~ 24 to ~ 26 h of a high carbohydrate diet and rehydration was undertaken as recovery.

**Results:**

During the entire RWL process, participants lost ~ 5.3% body mass. Body mass lost during the 2 h hot bath protocol was 2.17 ± 0.81 kg (~ 2.7% body mass) and 2.24 ± 0.64 kg (~ 2.8% body mass) for FWB and SWB, respectively (*P* = 0.647 between trials). Blood urea nitrogen, creatinine, sodium, chloride, hemoglobin, and hematocrit were increased (all *P* < 0.05), and plasma volume was decreased (~ 14%; *P* < 0.01), but did not differ between FWB and SWB, and were similar to baseline values after recovery. No indices of performance (e.g., countermovement jump, isometric strength, and functional threshold power) were impacted when RWL was followed by the recovery process.

**Conclusion:**

Under the conditions of this hot bath protocol, fluid loss was not augmented by the addition of ~ 5.0%wt/vol of Epsom salt during HWI, and RWL of ~ 5.3% body mass followed by > 24 h of recovery did not impact indices of performance.

## Introduction

In sports that have weight class restrictions, athletes attempting to ‘make weight’ frequently practice short-term weight loss, termed acute or rapid weight loss (RWL), in the ~ 72 h before weigh-in (Reale et al. 2017; Burke et al. 2021). Methods of RWL focus on the acute reduction of the contents of the gastrointestinal tract, and of total body water, through methods such as a low carbohydrate and low-residue diet, fluid restriction, and increasing sweat losses through exercise and/or heat exposure (Barley et al. 2018a; Hillier et al. 2019; Connor and Egan 2019). In mixed martial arts (MMA) athletes, combinations of these methods typically induce losses of ~ 5% to ~ 10% of body mass in the week before weigh-in (Coswig et al. 2015, 2019; Matthews and Nicholas 2017; Barley et al. 2018a; Brechney et al. 2019; Connor and Egan 2019; Hillier et al. 2019).

One method of heat exposure used to induce passive fluid loss is hot water immersion (HWI), which is often employed as part of weight-making strategies in combat sports (Pettersson et al. 2013; Matthews and Nicholas 2017; Brandt et al. 2018; Connor and Egan 2019; Kasper et al. 2019; Park et al. 2019; Gordon et al. 2021). Colloquially known as hot baths, this method typically involves short-duration HWI followed by ‘wrapping’ in warm clothing for a period of time before further exposures to HWI and wrapping (Connor et al. 2020; Kasper et al. 2019). The HWI is often performed in salt water, usually by the addition of Epsom salt, with the aim of augmenting the loss of body mass compared to that achieved by immersion in fresh water (Connor et al. 2020). Indeed, there is some empirical evidence for salt water immersion to augment fluid loss when compared to fresh water immersion (Whitehouse et al. 1932; Hertig et al. 1961; Hope et al. 2001), albeit these studies involve prolonged (≥ 3 h) HWI. The mechanistic basis for this effect is proposed as the addition of salt increasing the osmotic pressure difference between the immersion medium and body fluids, and/or attenuating the inhibitory effect on sweating that occurs during prolonged HWI, and thereby resulting in the greater fluid loss compared to fresh water (Whitehouse et al. 1932; Buettner 1953, 1959; Peiss et al. 1956; Hertig et al. 1961; Brebner and Kerslake 1964; Hope et al. 2001).

Our recent work has investigated a hot bath protocol incorporating short-duration (2 × 20 min) HWI with an Epsom salt concentration of ~ 1.6%wt/vol, but found no difference in body mass losses comparing fresh water and salt water immersion either at a fixed water temperature of 37.8 ºC (Connor et al. 2020), or when athletes self-adjusted the water temperature to the maximum temperature each could tolerate (~ 39.0 ºC) (Connor and Egan 2021). Our choice of a ~ 1.6% salt solution using Epsom salt was chosen for its ecological validity based on our knowledge of applied practice, and responses to a questionnaire in which fighters described typically the addition of 1 to 2 kg of Epsom salt to a standard sized bath (Connor et al. 2020). However, higher concentrations of salt, which would induce a larger osmotic gradient between the bath water and body fluids, may be required to augment fluid loss when compared to fresh water. For example, even in thermoneutral water, i.e., in the absence of sweating, immersion in a strong salt solution (either 11.5 or 20.0% salt as sodium chloride) induced passive fluid loss (Whitehouse et al. 1932). In water heated to 36/37 ºC, addition of 5% sodium chloride (~ 1709 mOsmol/kg) allowed for higher sweat rates during 3 h of immersion when compared to fresh water, with the effect more pronounced at salt concentrations of 10 and 15% (Hertig et al. 1961). Finally, immersion in seawater (~ 3.5% salt; ~ 1000 mOsmol/kg to ~ 1200 mOsmol/kg) resulted in ~ 32% greater loss of body mass compared to fresh water during 4 h of immersion at ~ 38 ºC (Hope et al. 2001). Given these observations, it may be that the concentration of salt in a hot bath should be at least 3.5% (Hope et al. 2001), or possibly greater (Buettner 1953, 1959; Hertig et al. 1961), if the aim is to augment the rate of passive fluid loss that would otherwise occur during HWI in fresh water.

Therefore, the present study investigated the magnitude of body mass losses in MMA athletes using a hot bath protocol with immersion in fresh water or salt water at a concentration of ~ 5%wt/vol of Epsom salt. Extending our previous work (Connor et al. 2020; Connor and Egan 2021), we also investigated the effects of ~ 28 to ~ 30 h of RWL on blood markers (plasma volume, kidney function, and electrolytes) and indices of performance. Hypohydration is well established as negatively impacting indices of performance (Savoie et al. 2015; Deshayes et al. 2020), and importantly, recent evidence suggests this to be the case even when major confounders such as expectancy effects because of a lack of blinding, and inadequate familiarization with methods of dehydration, are addressed (James et al. 2019). While RWL inherently involves dehydration processes, in professional combat sports such as MMA and boxing the competitive bout typically takes place ~ 30 to ~ 36 h after weigh-in (Burke et al. 2021). This time-period may allow for rehydration and recovery of muscle glycogen (Burke et al. 2021), but several studies have observed a residual negative impact on indices of performance even after ~ 16 to ~ 24 h of recovery (Oöpik et al. 1996; Moghaddami et al. 2016; Alves et al. 2018; Yang et al. 2018; Barley et al. 2018b; Kurylas et al. 2019). Therefore, we also investigated the effect of RWL followed by ~ 24 to ~ 26 h of recovery in the form of a high carbohydrate diet and rehydration on body mass, hydration status, blood markers, and indices of performance.

## Methods

### Participants

Thirteen male MMA athletes (29.5 ± 6.7 y; 1.81 ± 0.07 m; 83.0 ± 8.8 kg) with previous experience of RWL provided written informed consent to participate. The study was approved by the Human Research Ethics Committee of Dublin City University (permit number: DCUREC/2020/186). Participants comprised both amateur and professional fighters, but all participants were competing under professional weigh-in rules at the time of the study, i.e., weigh-in ≥ 24 h before competition. Each participant had previous experience of RWL, and the use of hot baths as part of that process, and each participant had made weight for competition on at least five occasions before participating in the study.

### Study design

**Fig. 1:**
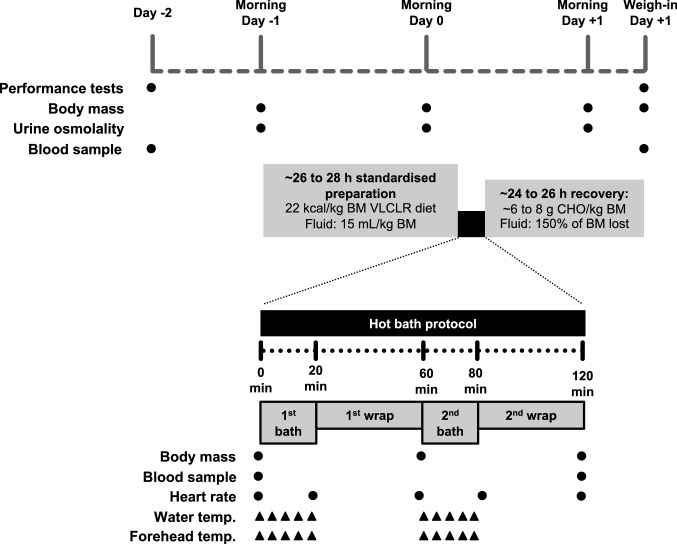
Study design schematic. Experimental trials were identical with the exception of the water condition in which they were immersed during the 1st and 2nd bath periods, and with these being fresh water bathing or salt water bathing on separate days. BM, body mass; CHO, carbohydrate; VLCLR, very low-carbohydrate, low residue

A crossover-repeated-measures design was employed to compare the effects on passive fluid loss of HWI using fresh water bathing (FWB) compared to salt water bathing (SWB). Participants performed two main experimental trials separated by at least 7 days, with the order of the FWB and SWB trials assigned in a counterbalanced manner, and participants randomized to which trial they performed first. The FWB and SWB trials were identical with the exception of the water condition in which they were immersed during the bathing periods (Fig. 1).

On Day − 2, a performance test battery was performed consisting of tests of leg power (countermovement jumps; CMJ), maximal strength (isometric hand-grip strength and isometric mid-thigh pull; IMTP), and a 3 min all-out exercise test on a cycle ergometer to estimate functional threshold power (FTP). At least 72 h before the first trial commencing, a familiarization session for the performance test battery was performed, which involved the participants undertaking the test battery in its entirety, and in an identical manner to that undertaken during the main trials.

On Day − 1 (the day before bathing), participants were prescribed to restrict fluid intake to 15 mL/kg of body mass, eliminate carbohydrate- and fiber-rich foods from their diet, and consume an energy intake of 22 kcal/kg of body mass, which was tracked using the MyFitnessPal mobile phone application (UnderArmour, USA). These practices are similar to what these participants routinely undertake to make weight for competition, and compliance with the prescribed protocol was confirmed verbally on Day 0.

On Day 0, participants arrived ~ 2 to ~ 4 h after waking to perform the bathing protocol. During this ~ 2 to ~ 4 h period, participants remained fasted and did not consume any fluids. Upon completion of the bathing protocol, the total body mass lost from Morning Day − 1 was calculated, and participants began the weight regain process by following the prescription to consume fluids (in L) to the equivalent to 150% of body mass lost (in kg) during the next 6 h (Sawka et al. 2007), and to consume 6 to 8 g/kg of body mass of carbohydrate during the rest of the day (Burke et al. 2021).

On Day + 1, participants were advised to follow their habitual fight day nutrition practices, and returned to undertake the performance test battery ~ 24 to ~ 26 h after completing the bathing protocol (Fig. 1). For their first trial, participants were asked to keep a record of what food and fluid they consumed from waking to before testing on both Day − 2 and Day + 1. For their second trial, participants were asked to repeat the timing and quantity of this intake for the respective days. Compliance with this approach was confirmed verbally upon arrival for testing on each day. To minimize the potential influence of circadian rhythms on indices of performance, the testing on Day + 1 was performed at the same time of day ± 1 h as performed on Day − 2. Participants completed their habitual training in the period between the main trials, but for the day before Day − 2, only low intensity training was allowed, and like dietary standardization was asked to be maintained consistent before each trial.

### Bathing protocol

The bathing protocol comprised of 20 min of HWI (“bath”) followed by 40 min wrapped in a rubberized sauna blanket (“wrap”). This 60 min bath and wrap protocol was repeated twice per main experimental trial, i.e., 2 h total, as described in our previous work (Connor et al. 2020; Connor and Egan 2021) (Fig. 1). One difference to these previous studies was that a sauna blanket (MiHIGH Infrared Sauna Blanket; MiHIGH Pty Ltd, Queensland, Australia) was used for the wrap periods, rather than a knitted wool hat, cotton t-shirt, hooded cotton sweatshirt, cotton tracksuit bottoms/sweatpants, and socks worn underneath several blankets in a heated bedroom. According to the manufacturer, the sauna blanket uses the same heating technology as an infrared sauna, emitting far infrared wavelengths.

For each bath, participants were submerged up to the neck for 20 min, i.e., head-out HWI. For the FWB trial, only fresh tap water was used in the bath. For the SWB trial, Epsom salt was added to the bath with 160 L capacity at a concentration of 6.25 kg in 125 L of water (i.e. ~ 5.0%wt/vol). Based on the chemical composition of Epsom salt (magnesium sulfate heptahydrate; MgSO_4_⋅7H_2_O), 5.0%wt/vol of Epsom salt would result in the osmolality of the salt water being ~ 406 mOsmol/kg.

Another difference to our previous work in the present study was that the initial water temperature of the bath was prepared to ~ 40.3 ºC rather than 37.8 ºC (Connor et al. 2020; Connor and Egan 2021), and participants maintained the water temperature at their maximum tolerable level, rather than self-adjusting the water temperature upwards as previously (Connor and Egan 2021). To maintain the water temperature, participants requested from the researchers the addition of boiling water from an electric kettle (1.5 L) to the bath ad libitum. The volume of additional boiling water per bath was noted. Additional salt was not added to adjust for the additional boiling water, and therefore, during the bathing process, the %wt/vol was estimated to decrease from 5.0 to ~ 4.7%, whereas the osmolality of the salt water was estimated to concomitantly decrease from ~ 406 to ~ 381 mOsmol/kg.

A floating thermometer (Avent Bath & Room Thermometer; Philips, UK) was checked at 4 min intervals for measurement of water temperature, but participants were not informed of the temperature during either bath or trial. At the same 4 min intervals, forehead temperature was measured as the mean of two measures using a digital infrared thermometer (Model HTD8813; LPOW, USA), whose range of precision according to the manufacturer’s instructions is ± 0.2 ºC. After 20 min of bathing, the participants exited the bath, briefly dried themselves with a towel before entering the sauna blanket for the next 40 min. Heart rate was measured using an automated heart rate and blood pressure monitor (UA-611; A&D Company Limited, Japan) immediately before and after the 1st bath, immediately before and after the 2nd bath, and immediately after the 2nd wrap.

### Body mass, urine, and blood sampling

Change in body mass, measured to the nearest 0.05 kg (model #63,667; Soehnle, Germany), was the primary outcome measure. Body mass was measured in minimal clothing, i.e., lower body short underwear in the form of briefs or boxer briefs, at several time-points: (i) upon waking on the day before bathing (Morning Day − 1), (ii) upon waking on the day of bathing (Morning Day 0), (iii) immediately before the 1st bath, (iv) immediately before the 2nd bath, (v) immediately after the 2nd wrap, (vi) upon waking on the day after bathing (Morning Day + 1), and finally (vii) on the day after bathing at “weigh-in” immediately to the performance test battery (Weigh-in Day + 1). Change in body mass induced by the entire RWL process, and whether a body mass deficit was present after recovery, were both calculated compared to body mass at Morning Day − 1.

Urine samples for the measurement of urine osmolality (Osmocheck Portable Osmometer; Vitech Scientific, UK) were taken upon waking on Day − 1, Day 0, and Day + 1. Participants were classified as hypohydrated using the criterion of urine osmolality of > 700 mOsmol/kg (Sawka et al. 2007).

Capillary blood was sampled at four time-points: (i) before undertaking the performance test battery on Day − 2, (ii) immediately before the 1st bath, (iii) immediately after the 2nd wrap, and finally (iv) before re-testing of performance on Day + 1. Participants were seated upright and stationary for ~ 3 min before a fingertip capillary blood sample (95 μL) was collected and analyzed for blood chemistry (glucose, blood urea nitrogen [BUN], creatinine, hematocrit, hemoglobin, the Anion Gap, sodium, potassium, chloride, ionized calcium, and total CO_2_) using the i-STAT 1 point-of-care handheld blood analyzer and CHEM8 + cartridges (Abbott Laboratories, USA) according to the manufacturer’s instructions. The CHEM8 + cartridges were the best available tool for point-of-care blood analysis, and our specific interest from this list of analytes were BUN and creatinine as indicators of acute kidney injury; sodium, potassium, and chloride as indicators of change in circulating electrolytes sensitive to sweat losses and dehydration; whereas the data for hemoglobin and hematocrit were used to calculate percentage change in plasma volume using the method of calculation described by Dill and Costill (1974). Due to technical issues resulting in missing data points, data for blood analysis are reported as *n* = 10 or *n* = 11 where appropriate.

### Performance test battery

The performance test battery was identical on Day − 2 and Day + 1. After arrival and having a capillary blood sample taken, participants performed a standardized general warm-up. First, 10 min of cycle ergometry (Wattbike Pro; Wattbike Ltd., Nottingham, England) at a cadence of > 70 rpm and a self-selected moderate intensity (rating of perceived exertion of 12–15). Next, bodyweight exercises consisting of five squats, five split-squats each side, five push-ups, and five CMJs were performed, after which lastly, another 5 min of cycle ergometry and the same bodyweight exercises were performed.

Leg power was measured by CMJ for which five jumps in total were performed with 10 s of rest taken between each jump. The participants were instructed to jump with maximal effort on each jump, and were required to keep the hands firmly placed on the hips throughout the jump. Jumps were performed on a dual-force plate system sampling at 500 Hz (Pasco PS-2141; Pasco Scientific Corp, USA) and CMJ height was calculated as previously described (Jordan et al. 2018). Data are reported as jump height (in cm) calculated as the average of three jumps after the worst and best jumps of the five attempts were excluded. The coefficient of variation (%CV) for this parameter was 5.7% in this cohort of athletes.

Isometric hand-grip strength test was measured using a hand-grip dynamometer (TKK 5401 Grip-D; Takei Scientific Instruments Co, Japan). The dynamometer was held at shoulder height to start and the participants were instructed to apply maximum force while lowering their arm to their side while in full elbow extension (Savva et al. 2013). Two maximum efforts per hand were performed by alternating each side, with the best score for each hand being recorded and averaged as a composite score. The %CV for this parameter was 5.0% in this cohort of athletes.

IMTP was performed in a customized power rack (Grip Ltd.; Ireland) standing on a dual-force plate system using a standardized protocol as previously described (Halperin et al. 2016). Participants were positioned in a body position similar to completing the second pull of a power clean with a flat trunk position and their shoulders in line with the bar. This position allowed participants to maintain a knee angle of ~ 120 to ~ 130°. Two 3 s IMTP efforts were performed applying 50 and 80% of perceived maximum effort. After these priming efforts, 30 s of rest was taken before completing the three 3 s maximal efforts separated by seated rest for 150 s. Data are reported as peak force given that this measure is the most reliable measure from the data output (Brady et al. 2020). The %CV for this parameter was 7.2% in this cohort of athletes.

FTP was estimated using the 3 min all-out test (Burnley et al. 2006) performed on an electromagnetically and air-braked cycle ergometer (Wattbike Pro; Woodway Inc., USA) (Wainwright et al. 2017), using a previously validated protocol (Hanson et al. 2019). Handlebar and saddle position/height were recorded during the familiarization visit and replicated for each subsequent testing day. The warm-up was standardized as 5 min of cycling at cadence of > 70 rpm and the same self-selected moderate intensity as above. The goal of this test is then to maintain the highest power output possible for the 3 min of effort. Cadence was kept between 90 and 110 rpm for the duration of the test. On conclusion of the test, maximum heart rate (via telemetry; Polar, Finland) and FTP were extracted for analysis. The %CV for maximum heart rate and FTP were 3.5 and 3.0%, respectively, in this cohort of athletes.

The smallest worthwhile difference (SWD) for each of the performance tests was set at 0.2 between-subject SD, which is suggested to represent a practically relevant change in performance in athletes (Hopkins et al. 2009). Thus, in this study, the SWD corresponded to 0.6 cm for CMJ height, 1.5 kg for hand-grip strength, 56 N for IMTP peak force, 2.3 bpm for maximum heart rate, and 5.1 W for FTP.

### Sample size calculation and early termination

The primary outcome was change in body mass as a consequence of the 2 h bath and wrap protocol. Therefore, a sample size calculation was performed (G*Power v.3.1) based on previous research demonstrating an effect of salt water to augment the magnitude of body mass lost during HWI when compared to fresh water (Hope et al. 2001). Using the body mass lost after 2 h of that 4 h immersion protocol, a time point analogous to the present work, and that being 0.98 ± 0.44 kg and 1.24 ± 0.80 kg for fresh water and salt water respectively, and an assumed correlation between conditions of 0.90, the required sample size to detect a difference between FWB and SWB at a Type I error rate (*α*) of 0.05 and a power (1-*β*) of 0.8 was *n* = 26. However, given the absence of effect in our previous research using a salt concentration of ~ 1.6% (Connor et al. 2020; Connor and Egan 2021), a priori we planned an interim data analysis for the assessment of futility, and therefore discontinuation, after completion of 50% of the required sample size, i.e., *n* = 13. In the absence of any difference between FWB and SWB for change in body mass with *n* = 13 (*P* = 0.647 between trials, *d* = 0.09; data reported below), we discontinued recruitment at that time.

### Statistical analysis

Statistical analysis and graphical representation were performed using GraphPad Prism v9.1 (GraphPad Software, Inc., USA). Normality of data was assessed with the Shapiro–Wilk normality test for which all data passed. All data are presented as mean ± SD. A two-way (condition*time) repeated-measures analysis of variance (ANOVA) was used to assess responses to the interventions for variables with serial measurements. A one-way repeated-measures ANOVA was used to assess whether an order effect was present in the indices of performance from Trial 1 to Trial 2 regardless of salt condition. When a main or interaction effect was observed, pairwise comparisons were performed with Bonferroni’s correction for which multiplicity-adjusted *P* values are reported. Paired *t* tests were used to assess differences between conditions for variables with two measurements, including to assess whether an order effect was present when comparing Trial 1 and Trial 2. The level of statistical significance for all tests was set at *P* < 0.05. Standardized differences in the mean were used to assess magnitudes of effects between conditions. These were calculated using Cohen’s *d* effect size and are interpreted using thresholds of < 0.2, ≥ 0.2, ≥ 0.5 and ≥ 0.8 for *trivial*, *small*, *moderate*, and *large*, respectively.

## Results

### Water temperature

**Fig. 2:**
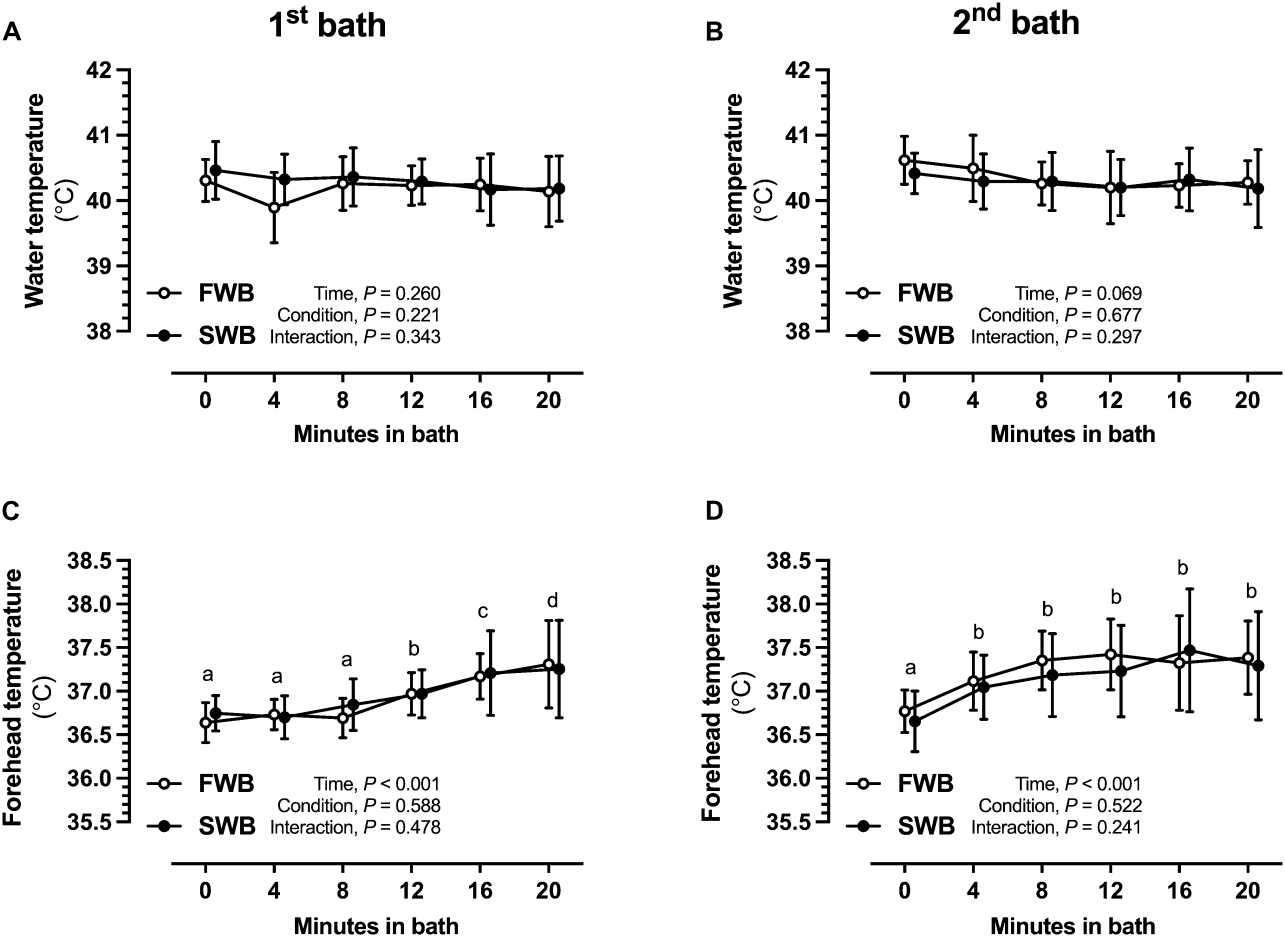
Water temperatures measured at 4 min intervals during each bath (**A** 1st bath; **B** 2nd bath) during experimental trials of fresh (FWB) or salt water (SWB), and forehead temperatures measured at 4 min intervals during each bath (**C** 1st bath; **D** 2nd bath). Data are mean values (*n* = 13, all male) with vertical bars representing SD

The starting water temperature did not differ between trials (1st bath, *P* = 0.374; 2nd bath, *P* = 0.133). The starting water temperature was 40.31 ± 0.32 ºC and 40.62 ± 0.37 ºC for the 1st and 2nd baths, respectively, in FWB (*P* = 0.240), and 40.46 ± 0.44 ºC and 40.42 ± 0.31 ºC for the 1st and 2nd baths, respectively, in SWB (*P* = 0.744). No interaction effect was observed for the effect of salt (1st bath, *P* = 0.343; 2nd bath, *P* = 0.297), and average water temperature remained above 40 ºC throughout the bathing periods (Fig. 2A and B). The volume of boiling kettle water added to each bath was 4.39 ± 1.14 L for FWB and 3.46 ± 1.42 L for SWB during the 1st bath of each trial (*P* = 0.055), and 2.31 ± 1.80 L for FWB and 2.65 ± 1.64 L for SWB during the 2nd bath of each trial (*P* = 0.513).

### Forehead temperature and heart rate response to the bathing protocols

**Fig. 3:**
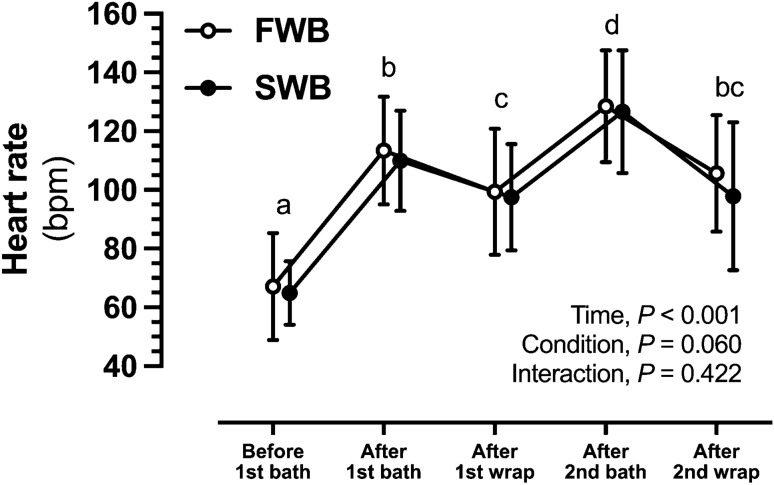
Heart rate responses to hot water immersion during experimental trials of fresh (FWB) or salt water (SWB). Data points are mean values (*n* = 13, all male) with vertical bars representing SD. Differences *within* conditions are noted by different letters representing significant differences (*P* < 0.05) between respective time-points, whereas time-points with the same letter are not different to each other

Forehead temperature increased in response to the hot bath protocol in both the 1st and 2nd bath periods (main effect of time, *P* < 0.001 for both) (Fig. 2C and D). Resting heart rate was similar for each trial before the 1st bath (FWB, 67 ± 18 bpm; SWB, 65 ± 11 bpm). Heart rate increased in response to the hot bath protocol (main effect of time, *P* < 0.001) and reached a measured peak of 128 ± 19 bpm and 127 ± 21 bpm after the 2nd bath period during FWB and SWB, respectively, but no main effect of condition (*P* = 0.166) or interaction effect (*P* = 0.762) was observed (Fig. 3).

### Changes in body mass

**Fig. 4:**
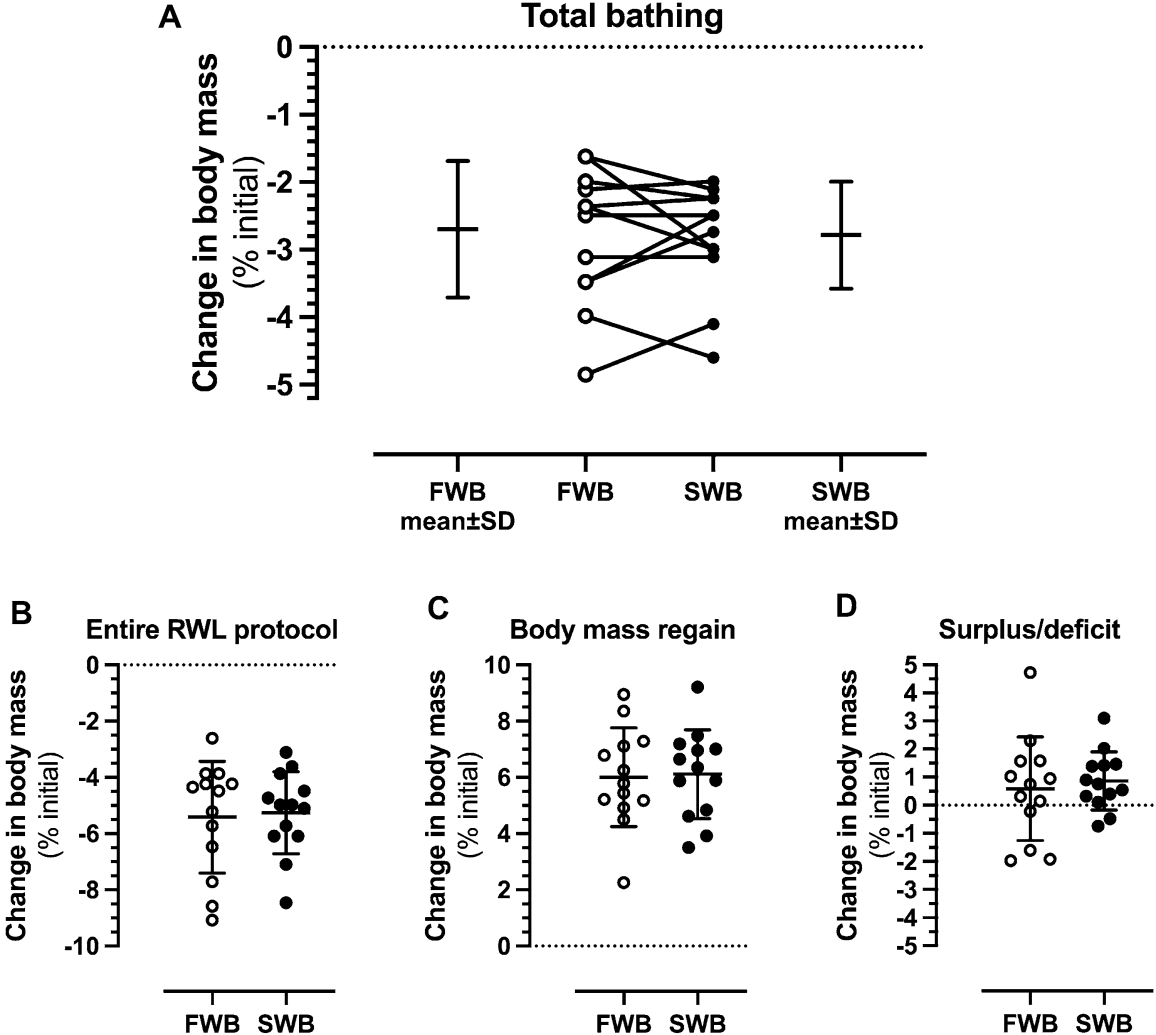
Percentage changes in body mass (relative to baseline recorded on Morning Day − 1) induced during **A** a hot bath protocol in fresh (FWB) or salt water (SWB) for a 2 h period comprising both baths and wraps, **B** the entire rapid weight loss (RWL) intervention, **C** the period of weight regain before weigh-in on Day + 1, and **D** as a measure of total body mass deficit or surplus at weigh-in on Day + 1 compared to Morning Day − 1. White (FWB) and black (SWB) circles in each panel represent individual data points. Mean values (*n* = 13, all male) are represented by the horizontal solid line with vertical bars representing SD for changes observed within each time-period that is defined above each panel

For change in body mass in absolute (kg) (Table 1) and relative (%initial body mass) (Fig. 4) terms, a main effect of time (*P* < 0.001), but neither a main effect of condition, nor a condition*time interaction effect, was observed. Similarly, there was no difference between conditions for changes in urine osmolality at the various time-points (Table 1).

**Table 1:** Body mass (kg) and hydration status assessed by urine osmolality (mOsmol/kg) at time-points during a rapid weight loss intervention featuring a hot bath protocol in fresh (FWB) or salt water (SWB)

	Morning Day − 1	Morning Day 0	Before 1st bath	After 1st bath & wrap	After 2nd bath & wrap	Morning Day + 1	Weigh-in Day + 1	*P* value
Body mass (kg)								Time, *P* < 0.001***
FWB	82.95 ± 8.78^a^	81.09 ± 7.89^b^	80.76 ± 7.79^b^	79.62 ± 7.70^c^	78.59 ± 7.64^d^	82.45 ± 7.83^a^	83.42 ± 7.84^a^	Condition, *P* = 0.754
SWB	82.86 ± 8.69^a^	81.16 ± 8.24^b^	80.88 ± 8.02^b^	79.70 ± 8.00^c^	78.64 ± 7.99^d^	82.73 ± 8.49^a^	83.56 ± 8.63^a^	Interaction, *P* = 0.655
Urine osmolality (mOsmol/kg)								Time, *P* = 0.002**
FWB	762 ± 217^a^	955 ± 145^b^				674 ± 269^a^		Condition, *P* = 0.570
SWB	695 ± 252^a^	845 ± 185^b^				769 ± 230^a^		Interaction, *P* = 0.067

Data are presented as mean ± SD, *n* = 13. Differences *within* conditions are noted by superscripted letters where different letters represent significant differences (*P* < 0.01) between respective time-points, whereas time-points with the same letter are not different to each other

**P < 0.01 and ***P < 0.001 for main and interaction effects from the two-way (condition*time) ANOVA analyses

Body mass losses induced by carbohydrate and fluid restriction were 2.18 ± 1.18 kg (*P* < 0.001; *d* = 0.26) and 1.98 ± 0.95 kg (*P* < 0.001; *d* = 0.24) in preparation for the FWB and SWB trials, respectively. These values represented losses of relative to initial body mass on Morning Day − 1 of 2.72 ± 1.47% and 2.47 ± 1.18% for the FWB and SWB protocols, respectively.

Body mass losses induced by the hot bath protocols were 2.17 ± 0.81 kg (*P* < 0.001; *d* = 0.28) and 2.24 ± 0.64 kg (*P* < 0.001; *d* = 0.28) for the FWB and SWB protocols, respectively, which corresponded to 2.70 ± 1.01% of initial body mass for FWB, and 2.78 ± 0.79% of initial body mass for SWB (Fig. 4A). Analysis for the presence of an order effect demonstrated no difference (*P* = 0.704) in body mass losses induced by the hot bath protocols when analyzed as Trial 1 (2.27 ± 0.71 kg) versus Trial 2 (2.14 ± 0.75 kg). FWB resulted in body mass loss of 1.15 ± 0.63 kg (*P* = 0.001; *d* = 0.15) during the 1st bath and wrap, and 1.02 ± 0.31 kg (*P* < 0.001; *d* = 0.13) during the 2nd bath and wrap. SWB resulted in body mass loss of 1.18 ± 0.25 kg (*P* < 0.001; *d* = 0.15) during the 1st bath and wrap, and 1.06 ± 0.45 kg (*P* < 0.001; *d* = 0.13) during the 2nd bath and wrap.

Total body mass losses induced by the entire RWL protocol were 4.35 ± 1.60 kg (*P* < 0.001; *d* = 0.53) and 4.22 ± 1.17 kg (*P* < 0.001; *d* = 0.51) for the FWB and SWB protocols, respectively. These values represented losses of relative to initial body mass on Morning Day − 1 of 5.42 ± 1.99% and 5.25 ± 1.46% for the FWB and SWB protocols, respectively (Fig. 4B).

On Morning Day − 1, 9 (FWB trial) and 7 (SWB trial) were classified as hypohydrated with a urine osmolality of > 700 mOsmol/kg (Sawka et al. 2007). On Morning Day + 1, 7 (FWB trial) and 9 (SWB trial) were classified as hypohydrated, and 8 (FWB trial) and 6 (SWB trial) participants were in a body mass deficit compared to Morning Day − 1. However, at Weigh-in Day + 1, i.e., before the performance test battery, only 4 (FWB trial) and 2 (SWB trial) participants were in a body mass deficit compared to Morning Day − 1. Overall, weight regain from the end of the 2nd wrap period to Weigh-in Day + 1 was 4.83 ± 1.41 kg (*P* < 0.001; *d* = 0.62) and 4.92 ± 1.27 kg (*P* < 0.001; *d* = 0.59) during recovery from the FWB and SWB protocols, respectively (shown as % of initial body mass in Fig. 4C), resulting in a body mass surplus compared to Morning Day − 1 of 0.47 ± 1.48 kg and 0.69 ± 0.83 kg, respectively (shown as % of initial body mass in Fig. 4D).

### Indices of performance

Across the five time-points measured (FAM, Trial 1 Day − 2, Trial 1 Day + 1, Trial 2 Day − 2, Trial 2 Day + 1), there was no order effect observed for any of indices of performance, i.e., CMJ height (*P* = 0.907), hand-grip strength (*P* = 0.722), IMTP peak force (*P* = 0.537), maximum heart rate (*P* = 0.284), and FTP (*P* = 0.874). Comparing between FWB and SWB trials, in the 3 min all-out test, the absence of interaction effects or main effects of time are indicative of there being no significant differences in FTP or maximum heart rate either between or within conditions (Table 2). Similarly, no differences in CMJ height, hand-grip strength, or IMTP peak force either between or within conditions were observed (Table 2).

**Table 2:** Countermovement jump (CMJ) height, hand-grip strength, isometric mid-thigh pull (IMTP) peak force, functional threshold power (FTP), and maximum heart rate (HR) measured before (Day − 2) and ~ 28 h after (Day + 1) a rapid weight loss intervention featuring a hot bath protocol in fresh (FWB) or salt water (SWB)

	FAM	Day − 2	Day + 1	*P* value
CMJ height (cm)	31.11 ± 4.36			Time, *P* = 0.572
FWB		32.93 ± 2.71	32.86 ± 3.30	Condition, *P* = 0.080
SWB		31.81 ± 3.86	32.29 ± 3.06	Interaction, *P* = 0.435
Hand-grip strength (kg)	46.7 ± 6.5			Time, *P* = 0.503
FWB		48.0 ± 6.8	48.9 ± 7.9	Condition, *P* = 0.739
SWB		48.8 ± 8.0	47.5 ± 7.6	Interaction, *P* = 0.066
IMTP peak force (N)	1766 ± 280			Time, *P* = 0.435
FWB		1690 ± 267	1757 ± 276	Condition, *P* = 0.785
SWB		1739 ± 288	1729 ± 287	Interaction, *P* = 0.152
3 min all-out test Maximum HR (bpm)	182.5 ± 9.4			Time, *P* = 0.374
FWB		183.5 ± 10.1	185.2 ± 11.4	Condition, *P* = 0.228
SWB		185.2 ± 12.4	186.1 ± 11.4	Interaction, *P* = 0.790
3 min all-out test FTP (W)	213.8 ± 21.0			Time, *P* = 0.642
FWB		219.6 ± 22.9	220.6 ± 26.4	Condition, *P* = 0.068
SWB		222.2 ± 25.4	223.7 ± 27.8	Interaction, *P* = 0.854

Data are presented as mean ± SD, *n* = 13. FAM, familiarization trial. Data for FAM are included for descriptive purposes. *P* values are obtained from two-way (condition*time) ANOVA analyses on the FWB and SWB data

### Blood markers

For blood markers, the absence of interaction effects or main effects of condition are indicative of there being no significant differences between conditions on these markers (Table 3). A main effect of time (all *P* ≤ 0.01) was observed for several markers (BUN, chloride, creatinine, hemoglobin, hematocrit, and sodium) with each being increased in blood samples taken immediately after the 2^nd^ wrap period, but returning to values similar to Day − 2 when measured after the period of recovery up to sampling at Weigh-in Day + 1 (Table 3). Declines in plasma volume induced by the entire RWL protocol were estimated as being -14.1 ± 12.1% for FWB (*P* = 0.003; *d* = 1.64) and -13.0 ± 11.4% for SWB (*P* = 0.004; *d* = 1.62).

**Table 3:** Blood markers measured at time-points during a rapid weight loss intervention featuring a hot bath protocol in fresh (FWB) or salt water (SWB)

	Pre-testing Day − 2	Before 1st bath	After 2nd bath & wrap	Weigh-in Day + 1	*P* value
Glucose (mg/dL)					Time, *P* = 0.041*
FWB	94.3 ± 9.2	88.1 ± 9.1	91.8 ± 14.8	100.2 ± 13.1	Condition, *P* = 0.290
SWB	94.8 ± 8.7	90.7 ± 6.1	104.9 ± 16.1	94.9 ± 11.5	Interaction, *P* = 0.024*
BUN (mg/dL)					Time, *P* < 0.001***
FWB	22.2 ± 4.4	26.9 ± 6.9^a^	27.8 ± 7.3^a^	20.9 ± 6.6	Condition, *P* = 0.030*
SWB	18.8 ± 5.2	25.5 ± 7.4^a^	27.3 ± 6.7^aa^	20.0 ± 5.8	Interaction, *P* = 0.481
Creatinine (mg/dL)					Time, *P* < 0.001***
FWB	0.99 ± 0.16	1.03 ± 0.14	1.19 ± 0.23^a^	1.13 ± 0.18	Condition, *P* = 0.125
SWB	0.96 ± 0.11	1.00 ± 0.11	1.20 ± 0.12^aaa^	0.94 ± 0.10	Interaction, *P* = 0.071
Hematocrit (%)					Time, *P* = 0.001**
FWB	44.2 ± 3.7	45.5 ± 3.1	48.2 ± 4.0^a^	45.1 ± 3.4	Condition, *P* = 0.517
SWB	44.3 ± 3.2	46.2 ± 2.1	47.9 ± 2.3^a^	46.1 ± 2.7	Interaction, *P* = 0.644
Hemoglobin (g/L)					Time, *P* = 0.001**
FWB	15.0 ± 1.3	15.5 ± 1.1	16.4 ± 1.4^a^	15.3 ± 1.2	Condition, *P* = 0.536
SWB	15.1 ± 1.1	15.7 ± 0.7	16.3 ± 0.8^a^	15.7 ± 0.9	Interaction, *P* = 0.640
Change in plasma volume (%)					Time, *P* = 0.001**
FWB		– 5.1 ± 7.4	– 14.1 ± 12.1^a^	2.7 ± 13.6	Condition, *P* = 0.630
SWB		– 7.0 ± 10.0	– 13.0 ± 11.4^a^	– 6.3 ± 12.8	Interaction, *P* = 0.657
Anion gap (mM)					Time, *P* = 0.805
FWB	16.4 ± 1.8	16.4 ± 2.7	16.0 ± 2.4	15.2 ± 2.8	Condition, *P* = 0.851
SWB	15.7 ± 1.6	15.7 ± 2.1	16.3 ± 2.6	16.6 ± 1.9	Interaction, *P* = 0.173
Sodium (mM)					Time, *P* < 0.001***
FWB	139.9 ± 2.2	141.3 ± 1.6^a^	143.5 ± 2.8^aa,b^	139.7 ± 1.7	Condition, *P* = 0.361
SWB	140.5 ± 1.5	141.3 ± 1.3	144.1 ± 2.5^a,bb^	140.1 ± 1.8	Interaction, *P* = 0.814
Potassium (mM)					Time, *P* = 0.642
FWB	4.63 ± 0.43	4.87 ± 0.37	4.75 ± 0.39	4.67 ± 0.41	Condition, *P* = 0.227
SWB	4.82 ± 0.36	4.80 ± 0.27	4.80 ± 0.32	4.90 ± 0.24	Interaction, *P* = 0.272
Chloride (mM)					Time, *P* < 0.001***
FWB	103.6 ± 2.7	105.5 ± 2.5	108.3 ± 3.1^a,bbb^	103.6 ± 1.6	Condition, *P* = 0.547
SWB	103.6 ± 1.6	105.5 ± 1.5 ^a^	107.8 ± 2.9^a^	102.7 ± 1.7	Interaction, *P* = 0.507
iCalcium (mM)					Time, *P* = 0.311
FWB	1.25 ± 0.07	1.29 ± 0.08	1.29 ± 0.07	1.35 ± 0.23	Condition, *P* = 0.983
SWB	1.29 ± 0.07	1.26 ± 0.08	1.32 ± 0.10	1.31 ± 0.07	Interaction, *P* = 0.583
Total CO_2_ (mM)					Time, *P* = 0.125
FWB	25.5 ± 1.6	25.7 ± 2.3	25.3 ± 1.6	26.5 ± 2.2	Condition, *P* = 0.283
SWB	27.1 ± 1.6	25.8 ± 1.2	25.6 ± 1.4	26.6 ± 1.6	Interaction, *P* = 0.334

Data are presented as mean ± SD, *n* = 10 or 11

*BUN* blood urea nitrogen, *CO*_*2*_ carbon dioxide

*P < 0.05; **P < 0.01; ***P < 0.001 for main and interaction effects from the two-way (condition*time) ANOVA. Where a main effect of time was indicated, differences *within* conditions are noted by ^a^*P* < 0.05, ^aa^*P* < 0.01, and ^aaa^*P* < 0.001 compared to Pre-testing Day − 2, and ^b^*P* < 0.05, ^bb^*P* < 0.01, and ^bbb^*P* < 0.001 compared to Before 1st bath

## Discussion

Given that our previous work using ~ 1.6% salt solutions did not reveal an effect of salt to augment body mass loss during a hot bath protocol (Connor et al. 2020; Connor and Egan 2021), the present study investigated body mass losses when the salt concentration is increased to ~ 5.0%wt/vol. This higher concentration is more similar to immersion studies where an effect of salt to augment the loss of fluid and/or body mass has been observed (Whitehouse et al. 1932; Hertig et al. 1961; Hope et al. 2001). However, the present study demonstrates that the body mass lost during a hot bath protocol using fresh water (FWB) is similar to a protocol using ~ 5.0%wt/vol of Epsom salt (SWB).

Body mass losses induced by ~ 26 to ~ 28 h of restriction of fluid intake combined with a low-residue, low-carbohydrate diet were ~ 2.6% of body mass. This is similar in magnitude to the suggestion of ~ 3% reduction to be expected by short-duration restriction of carbohydrate and fluid, and emptying of the gastrointestinal contents using a low-residue diet (Reale et al. 2017; Burke et al. 2021), and is also similar to our previous work (Connor et al. 2020; Connor and Egan 2021). However, the percentage of body mass lost during the entire RWL process was greater in the present study at ~ 5.3% compared to those previous studies where ~ 4.3% (Connor et al. 2020) and ~ 4.5% (Connor and Egan 2021) were observed. The larger magnitude is explained by differences in percentage of body mass lost in the bathing protocol, which was ~ 2.7% loss of body mass in this study compared to ~ 2.1% in the other studies. This difference may simply reflect inter-individual differences in RWL between studies. Alternatively, commencing bath in water of higher temperature (e.g. ~ 40.3 ºC versus 37.8 ºC), and using a sauna blanket for the wrap periods rather than cotton clothing in a warm room, may result in more efficient loss of body mass per unit of time invested in such a protocol.

Together, these findings across three studies suggest that the addition of salt to HWI does not augment the loss of body mass compared to fresh water, at least in the hot bath protocol employed. The caveat that this conclusion only applies to the hot bath protocol employed is important, because several prior studies do indeed demonstrate an effect of salt to augment immersion-induced loss of fluid and/or body mass in various experimental models including whole-body immersion, and localized immersion of an arm/hand or leg/foot (Whitehouse et al. 1932; Buettner 1953, 1959; Peiss et al. 1956; Hertig et al. 1961; Brebner and Kerslake 1964; Hope et al. 2001). There are two suggested mechanisms for this phenomenon. First, that during immersion in salt water, the osmotic pressure difference between the immersion medium and body fluids results in greater fluid loss compared to fresh water, and/or second, that salt water serves to attenuate an inhibitory influence on the decline in sweat rate that usually occurs with prolonged immersion in hot fresh water (Whitehouse et al. 1932; Buettner 1953, 1959; Peiss et al. 1956; Hertig et al. 1961; Brebner and Kerslake 1964; Hope et al. 2001).

The absence of an effect of salt in our work may be explained by duration of immersion being much shorter than those previous studies observing an effect. For example, those studies have used immersion times of 3 h (Hertig et al. 1961), 4 h (Hope et al. 2001) and 5 h (Whitehouse et al. 1932; Brebner and Kerslake 1964). Despite our protocol comprising of 2 h of passive heating, HWI only accounts for 2 × 20 min of this time-period. Hope et al. (2001) observed a difference of ~ 600 g of body mass lost in 4 h when comparing immersion in fresh water to salt water (sea water) at 38ºC (Hope et al. 2001). This difference between conditions would be the equivalent of ~ 2.5 g per minute assuming linearity in the response. Translating this rate into our 40 min of total time spent immersed in water would result in an expected difference of just 100 g between FWB and SWB. Therefore, for the addition of salt to have the desired impact of augmenting loss of body mass through passive fluid loss, much longer immersion times than the 2 × 20 min employed in this study may be required.

Another consideration, however, is the osmolality of the salt water given the proposed mechanism around the osmotic pressure difference between the immersion medium and body fluids. While the %wt/vol of salt is most commonly used as the descriptor of the salt water condition, the osmolality will be a function of both the concentration and type of salt. Our previous work using 1.6%wt/vol of Epsom salt (Connor et al. 2020; Connor and Egan 2021), and the present study using 5.0%wt/vol of Epsom salt, would result in an osmolality of ~ 130 and ~ 406 mOsmol/kg, respectively, which would decline somewhat with the addition of boiling water to maintain or increase the water temperature while bathing. Thus, these salt water baths were, respectively, hypotonic and only mildly hypertonic relative to the osmolality of body fluids (i.e., ~ 280 to ~ 295 mOsmol/kg). In contrast, when augmented body mass losses have been previously observed, these salt water baths were markedly hypertonic, i.e., 5%wt/vol of sodium chloride (Hertig et al. 1961) being ~ 1709 mOsmol/kg, and seawater (Hope et al. 2001) being ~ 3.5% salt and ~ 1000 to ~ 1200 mOsmol/kg. Therefore, while Epsom salt was used for its ecological validity, a salt such as sodium chloride may be more effective on a %wt/vol basis. Alternatively, Epsom salt would need to be used at > 12.3%wt/vol to produce an osmolality of > 1000 mOsmol/kg. These points assume that the osmotic gradient is an important mechanism by which salt water augments loss of body mass during HWI, and tentatively suggest that > 1000 mOsmol/kg is a valid threshold above which these effects would be observed.

The present study extends our previous work by measuring heart rate during the hot bath protocol, and measuring changes in blood markers during the RWL process, in addition to investigating effects of the RWL followed by ~ 24 to ~ 26 h of recovery on indices of performance. The heart rate data during the hot bath protocol demonstrate that a moderate degree of cardiovascular stress was induced as indicated by heart rate averaging ~ 110 bpm throughout the 2 h period and a measured peak at 128 ± 19 bpm and 127 ± 21 bpm during FWB and SWB, respectively. These values are equivalent to ~ 68% of the participants’ age-predicted maximum heart rate.

The concentrations of several analytes in blood were increased during the hot bath protocol. Specifically, in blood samples taken immediately after the 2nd wrap period, concentrations of BUN, chloride, creatinine, hemoglobin, and sodium were each increased, as was the hematocrit value, but each returned to values similar to baseline by weigh-in on Day + 1. Calculation of plasma volume from hemoglobin and hematocrit revealed an average decrease in plasma volume induced by RWL of ~ 14% when measured upon completion of the 2nd wrap. This value is somewhat greater than that observed by (Hope et al. 2001) of ~ 7 to ~ 12%, but perhaps unsurprising given that the overall loss of body mass during the RWL protocol in the present study was approximately double of that previous work. In contrast, when elite amateur boxers undertook RWL in which a similar quantity of body mass was lost (5.6 ± 1.7%), the reduction in plasma volume was smaller at 8.6 ± 3.9% (Reljic et al. 2013). In that study, the RWL process was over a 5 day period, which potentially suggests that a shorter time frame of RWL and/or exposure to HWI may lead to greater loss of plasma volume or differential effects on different compartments of body water. There are two caveats that apply to the interpretation of these data for plasma volume. First, the i-STAT blood analyzer derives the value for hemoglobin using a proportionality constant after the measurement of hematocrit by a conductometric method, so the plasma volume data are based on an estimation of hemoglobin rather than direct measurement. Alternatively, using hematocrit only and thereby calculating loss of blood volume (Dill and Costill 1974), RWL resulted in a decrease in blood volume by ~ 8% in both conditions upon completion of the 2nd wrap. Second, postural changes are known to acutely influence measures of plasma volume (Pivarnik et al. 1986; Lippi et al. 2015), and a reduction in plasma volume of ~ 4.8% was previously observed within the initial 5 min after moving from a supine to seated (Pivarnik et al. 1986). Although the seated posture and rest period was consistent before blood sampling on Day − 2, before the 1st bath on Day 0, and on Day + 1, the sample taken upon completion of the 2nd wrap was preceded by 40 min in a supine position and only ~ 3 min of equilibration in a seated position. Therefore, the change in posture from a supine to seated position may have also contributed to decrease in plasma volume observed in response to the hot bath protocol.

Also of note is the observation of increased BUN and creatinine concentrations as these are often used as biomarkers of acute kidney injury (AKI) (Edelstein 2008; Kellum and Lameire 2013; Ostermann et al. 2020). RWL of > 4% of body mass consistently results in an increase in BUN and creatinine, which has been suggested as an indication of AKI being caused by RWL (Lakicevic et al. 2021). AKI has been previously defined as an increase in serum creatinine concentration by ≥ 0.3 mg/dL within 48 h (Kellum and Lameire 2013), a threshold which just two of our participants exceeded and which occurred within the 2 h bathing period. Additionally, the utility of circulating BUN and creatinine concentrations as sensitive and specific markers of AKI has been questioned (Edelstein 2008; Ostermann et al. 2020), whereas traditional measures of AKI are limited in their ability to classify AKI during heat stress, especially when combined with dehydration and/or exercise (Chapman et al. 2021). BUN and creatinine concentrations are indirect measures of AKI rather than direct measures of tissue injury such as with creatine kinase and cardiac troponin from skeletal muscle and heart, respectively. Direct measures of AKI in the circulation remain to be firmly established, especially those that can differentiate between ‘pre-renal’ and ‘intrinsic’ causes of change in circulating markers (Edelstein 2008; Ostermann et al. 2020; Chapman et al. 2021). Moreover, changes in BUN and creatinine concentrations are generally delayed in their response to AKI rather than acutely responsive (Edelstein 2008; Ostermann et al. 2020). Our data indicate an acute response in creatinine concentration to increase over the 2 h bathing period, whereas BUN concentration was already increased after the diet and fluid restriction, and increased further during bathing. Hence, it remains unclear whether these increases can indeed be considered to be evidence of AKI, or whether these simply reflect the well-established hemoconcentration effect of an acute decrease in plasma volume (Harrison 1985). In favor of the former is that it is well established that heat stress, especially when combined with physical exertion, can result in AKI (Chapman et al. 2021). Especially relevant to the present study is that heat stress-associated AKI is also influenced by hydrostatic pressure of water when HWI is used to apply the heat stress in experimental contexts (Chapman et al. 2021). Therefore, changes in markers of AKI during RWL and comprehensive assessment of kidney function should continue to be investigated by future research to better understand this phenomenon given its implications for the welfare of athletes who repeatedly undertake RWL.

Immediately before the performance testing on Day + 1 represented a ~ 24 to ~ 26 h recovery period at which point only 4 (FWB trial) and 2 (SWB trial) participants remained in a body mass deficit compared to Morning Day − 1. On average, there was a body mass *surplus* of 0.47 ± 1.48 kg and 0.69 ± 0.83 kg compared to Morning Day − 1 after recovery from FWB and SWB, respectively. This surplus is in contrast to the deficit observed on average in our previous work (Connor et al. 2020; Connor and Egan 2021), but is explained by the ~ 2 to ~ 4 h longer recovery time in the present study due to the inclusion of the performance tests. Therefore, despite the loss of ~ 5.3% of body mass in ~ 28 to ~ 30 h, blood markers had returned to values similar to baseline after ~ 24 to ~ 26 h of recovery. In practice, the time from weigh-in until official competition in professional MMA is usually longer, i.e., ~ 30 to ~ 36 h, but even with a longer time-period for rehydration, the majority of MMA athletes have been observed to be hypohydrated up to 2 h before competition (Jetton et al. 2013; Matthews and Nicholas 2017). Based on these observations, regain of body mass alone was suggested as potentially not being a good indicator of returning to a euhydrated state, but there is some debate about the validity of the classification of hypohydration through assessment of hydration status by spot analysis with urine measures (Cheuvront et al. 2015; Barley et al. 2020). For example, an alternative to the criterion of urine osmolality of > 700 mOsmol/kg being classified as hypohydration (Sawka et al. 2007) has been proposed as ≥ 925 mOsmol/kg (Armstrong et al. 2010). Using the Armstrong et al.’s threshold, only 3 (FWB trial) and 3 (SWB trial) participants were classified at hypohydrated at Morning Day + 1 compared to 7 (FWB trial) and 9 (SWB trial) using the Sawka et al.’s threshold.

No indices of performance were impacted by the RWL and recovery process when compared to pre-RWL values in either the FWB or SWB conditions. These results are in contrast to studies that have demonstrated a residual negative impact on indices of performance after ~ 24 h of recovery (Alves et al. 2018; Barley et al. 2018b; Kurylas et al. 2019). In one study, athletes were dehydrated by ~ 5% of body mass through exercise in a heated room, and performance tests were completed 3 and 24 h after the intervention (Barley et al. 2018b). Vertical jump was unaffected by dehydration and recovery; hand-grip strength was weaker at 3 but not 24 h; medicine ball chest throw distance was shorter at 24 h, but not 3 h; and repeated sled push performance was worse at both 3 and 24 h after dehydration (Barley et al. 2018b). Therefore, there are likely to be time course-specific effects on performance in response to RWL and recovery, and which may also be impacted by whether passive or active methods of dehydration are employed, and the choice of performance test. Active methods of dehydration, i.e., involving exercise, may lead to residual fatigue and depletion of energy stores (Savoie et al. 2015), and can produce divergent responses in relation to changes in plasma volume, serum and urine osmolality, and performance, compared to passive dehydration (Nielson et al. 1981; Caldwell et al. 1984; Muñoz et al. 2013). Moreover, if a chosen performance test is not sensitive enough to detect physiological and performance changes, if any, that may be happening in response to RWL, the conclusion that there are no negative performance consequences of RWL when followed by adequate recovery and rehydration may be a type II error, i.e., false-negative finding.

Relatedly, this study was powered using the primary outcome of change in body mass as a consequence of the 2 h bath and wrap protocol. Given the absence of effect in our previous research using a salt concentration of ~ 1.6% (Connor et al. 2020; Connor and Egan 2021), like our previous approach (Connor and Egan 2021) a priori we planned an interim data analysis for the assessment of futility, and therefore discontinuation. However, the sample size was based on data derived from pre-to-post differences in a crossover design, and therefore, it is likely that the sample size is underpowered for the analysis of serial time point data such as those analyzed by ANOVA. In this scenario, again a type II error for observing the lack of differences between FWB and SWB cannot be fully discounted. Additionally, there are several methodological limitations that could be addressed in future work including the measurement of body temperature with a valid measure of core temperature (either esophageal or rectal), and the inclusion of a body mass measurement immediately after each period of HWI to isolate the effects of salt versus fresh water during HWI specifically rather than the entire hot bath protocol including wrapping periods.

In summary, short-duration HWI combined with periods under an infrared sauna blanket is an effective method of RWL to induce a loss of ~ 2.7% of body mass during 2 h of bathing (2 × 20 min) and wrapping (2 × 40 min). Using this protocol, the total amount of body mass lost when the water was supplemented with ~ 5.0%wt/vol of Epsom salt was similar to fresh water. When an appropriate refueling and rehydration strategy was followed, the ~ 5.3% loss of body mass during the overall ~ 28 to ~ 30 h RWL period was not detrimental in terms of blood markers or indices of performance measured after the ~ 24 to ~ 26 h recovery period.

## Abbreviations

AKI: Acute kidney injury
BUN: Blood urea nitrogen
CMJ: Countermovement jumps
FTP: Functional threshold power
FWB: Fresh water bathing
HWI: Hot water immersion
IMTP: Isometric mid-thigh pull
MMA: Mixed martial arts
RWL: Rapid weight loss
SWB: Salt water bathing
